# Robust superhydrophobic surface on Al substrate with durability, corrosion resistance and ice-phobicity

**DOI:** 10.1038/srep20933

**Published:** 2016-02-08

**Authors:** Guoyong Wang, Shuai Liu, Sufeng Wei, Yan Liu, Jianshe Lian, Qing Jiang

**Affiliations:** 1 Key Laboratory of Automobile Materials, Department of Materials Science and Engineering, Jilin University, No. 5988 Renmin Street, Changchun 130025, PR China; 2 Key Laboratory of Advanced Structural Materials, Changchun University of Technology, Changchun 130012, P.R. China; 3 Key Laboratory of Bionic Engineering (Ministry of Education) and State Key Laboratory of Automotive Simulation and Control, Jilin University, Changchun 130022, China

## Abstract

Practical application of superhydrophobic surfaces is limited by the fragility of nanoscale asperities. Combining chemical etching and anodization, microscale pits and nanoscale pores, instead of the micro and nano protrusions on traditional superhydrophobic surfaces mimicking Lutos leaves, were fabricated on commercially pure aluminum surfaces. After modified by FDTS, the surfaces were superhydrophobic and self-cleaning. The ultrahigh hardness and electrochemical stability of Al_2_O_3_ coating endowed the surface excellent mechanical durability and good corrosion resistance. Because the method is scalable, it may find practical application on body panels of automobiles and aircrafts and so on.

 As a water contact angle on a solid surface is greater than 150°, the surface is named superhydrophobic surface(SHS)^1^,^2^. The interest in SHS has grown exponentially over recent decades^3^. Since the lotus leaf dual hierarchical structure was discovered, researchers have investigated the foundations of self-cleaning behaviour^1^. Actually, it has been found out that the micro/nanostuctures combining with low surface energy bring superhydrophobic properties^1^. SHSs are usually classified as slippery surfaces (heterogeneous wetting state or Cassie-Baxter state) or sticky surfaces (homogeneous wetting state or Wenzel state). On a slippery surface, bouncing water droplets do not penetrate into the asperities of the surface. Most part of the droplet is separated by an air cushion. So the interaction between the droplet and the surface is lowered. That endows the surface with self-cleaning properties. An effortless rolling off of water droplets always can take along the contaminations. A sticky surface also exhibits a high water contact angle (>150°), and high roughness but water droplets are less prone to roll off from it. In that case, water droplets can penetrate into cavities of the surface. Thus, the high roughness increases the contact area, and as consequence, the liquid-solid interactions are increased^1^,^2^,^4^,^5^. Slippery surfaces are important in many industrial and biological processes, such as the prevention of adhesion of fogging and snow and ice (ice-phobic)^6^,^7^, self-cleaning^8^,^9^,^10^,^11^, reduction of frictional drag on ship hulls^12^, buoyancy^13^,^14^,^15^, corrosion-resistant^16^,^17^, oil-water separation^18^,^19^,^20^,^21^, metal refining, stain-resistant textiles^22^,^23^, and cell mobility^24^. In past decades, there have been many attempts at preparing artificial self-cleaning surfaces by mimicking lotus leaf surface structure in different ways. These surfaces have approached extremely high contact angle, even close to 180°, and very low contact angle hysteresis^9^,^11^,^15^,^25^,^26^. However, these surfaces are still far from application because the asperities of surface, especially the nanoscaled wires/tubes/protrusions which are vital for slippery surfaces, are very susceptible to the application environment, such as abrasive wear, and corrosive medium^27^,^28^. Meanwhile, these surfaces are generally made by controlling the surface chemistry and surface roughness of various expensive materials, which are then applied by means of complex time-consuming processes.

Recently, many methods have been developed to tackle the durable problems. The basic motivation is to seek a way to mitigate the degradation of non-wetting surfaces, as illustrated by Fig. 1^24^. For example, Wong *et al.* developed a robust SHS by introducing two-tier roughness which combined microcone and nanograss structure on silicon^29^. The strong microcones served as pillars especially when the cones were truncated as the pressure was high. Thus the beneath nanograss was carefully protected. Takhiro *et al.* coated a superhydrophobic Cerium Oxide film on magnesium alloy to increase the durability in corrosive NaCl aqueous solution^30^,^31^. Yong *et al.* developed CNT composite structures by replication of a micropatterned silicon surface using an epoxy resin and by deposition of the CNT composite using a spray method^32^. Tanmoy Maitra *et al.* fabricated robust hierachically nanotextured SHSs on aluminium. The surface design and development were based on control of microscale morphologies through aluminium etching and nanoscale morphologies including a self-assembled FDTS monolayer, PDMS thin layers, and MTS nanofibers^33^. All these methods involve robust microroughness that provides protection to a more fragile nanoscale roughness^34^,^35^. The microroughness can be optimized with mechanical stability in mind while the residual nanoroughness after abrasion ensures non-wettability.

In this paper, artificial structured superhydrophobic and self-cleaning surface has been produced on aluminium substrate using an industry compatible approach. In this approach which is illustrated in Fig. 1b, the microscale asperities were constructed by millions of etching sharp pits on the aluminium substrate, and the nanoscale asperities consisted of millions of nanopores induced by high field anodization, instead of the nanowires on Lotus leaves^36^,^37^,^38^. For the very fragile nanoscale protrusions are absent in the present approach, the synthesized surface has very good mechanical durability and corrosion resistance and the resistance to liquid meniscus impalement under drop impact and high-pressure underwater environments and ice-phobicity. For the approach is scalable and low-cost, it should have a prospective application in industry.

## Experimental Details

### Dual-scale roughness fabrication

Polycrystalline aluminum plates (GB 1A99, Libaida Ltd. China) with a dimension of 5 cm × 2 cm × 0.2 cm were selected as substrate. The plates were cleaned ultrasonically in sequence with alcohol and deionized water for 5 min, respectively. And then, they were immerged in NaOH aqueous solution (3% wt) heating in water bath at a temperature of 70 °C for 5 min to remove the oxidation layer and residual oil. Firstly, the fresh plates were etched in 3 M HCl aqueous solution (Beijing Chemical Works) for 11 min at room temperature to form multiple facets. Secondly, the porous alumina layers were created on the multiple facets by anodization at a constant current density of 6.25 A/dm^2^ for different duration in 2 M sulphuric acid aqueous solution at room temperature. Finally, the modification was performed by dip-coating in 0.5% 1H, 1H, 2H, 2H-perfluorooctadecyltrichlorosilane (FDTS) (Sigma-Aldrich) ethanol solution for 24 h and curing at 100 °C for 2 h.

### Characterization

Water contact angles (CAs) and sliding angles (SAs) were determined via a CCD camera on an OCA20 measurement system (Dataphysics GmbH, Germany), which was equipped with a rotating sample stage, with a 5-μL droplet of deionized water at ambient temperature. All measurements were repeated five times on different sample spots. SEM images were taken by field emission scanning electron microscopy (FESEM, JEOL JSM-6700F, Japan) at an electron acceleration voltage of 8 kV. X-ray photoelectron spectroscopic (XPS) tests were conducted with a surface analyzer (Kratos Axis Ultra DLD) using an Al Kα source working at 14 kV and 25 mA. All digital images and digital videos were recorded by a camera of Nikon D5000 (Japan). The anti-icing properties were investigated in natural environment. The hardness of the anodized sample and the substrate was investigated on a nanoindentation (G200, Agilent, USA) with a Berkovich tip. The maximum depth was 500 nm for the anodized sample in order to accurately evaluate the hardness of the thin alumina layer. The maximum depth was 1000 nm for the substrate. Then the force was unloaded to 10% the maximum load and held constant for 20 s for the thermal drift calibration.

### Mechanical durability tests

The mechanical durability of the SHS was tested by sand abrasion and polypropylene fabric abrasion, respectively. Sands with diameter ranging from 120 μm to 260 μm flowed down onto the surface at a speed of 9 g/min from a height of 30 cm for 20 min. The CAs and SAs were measured after every two minutes sand impacting. For the polypropylene fabric abrasion, the surface was pressed on a polypropylene fabric strap by a weight of 50 g which can generate a pressure of 500 Pa on the surface and wiped along the strap at a speed of 3.62 cm/s for 4 m. The CAs and SAs were measured after every 20 cm long wiping. The water-impacting test was carried out using the reported method^39^: the SHS was impacted by 25 μl droplets falling from 20 cm. The sample was placed at a SA of 45°.

### Corrosion resistance tests

All electrochemical measurements were performed in 3.5 wt% NaCl aqueous solution (PH = 6.2) at room temperature, using a computer-controlled potentiostat (Netherlands, IVIUMSTAT) under open circuit conditions. The testing samples and a platinum plate were used as the working and counter electrodes, respectively. The working electrode was covered by epoxy with a cubic window, the area of which was 1 cm^2^. A saturatued calomel electrode (SCE) was used as the reference electrode. The reference electrode was set in the vicinity of the cubic window. The testing samples were immersed in the NaCl solution for 10 min, allowing the system to be stabilized, and potentiodynamic polarization curves were subsequently measured with respect to the OCP at a scanning rate of 50 mV/s from −1.8 V to +0.2 V.

## Results and Discussion

**Figure 2:**
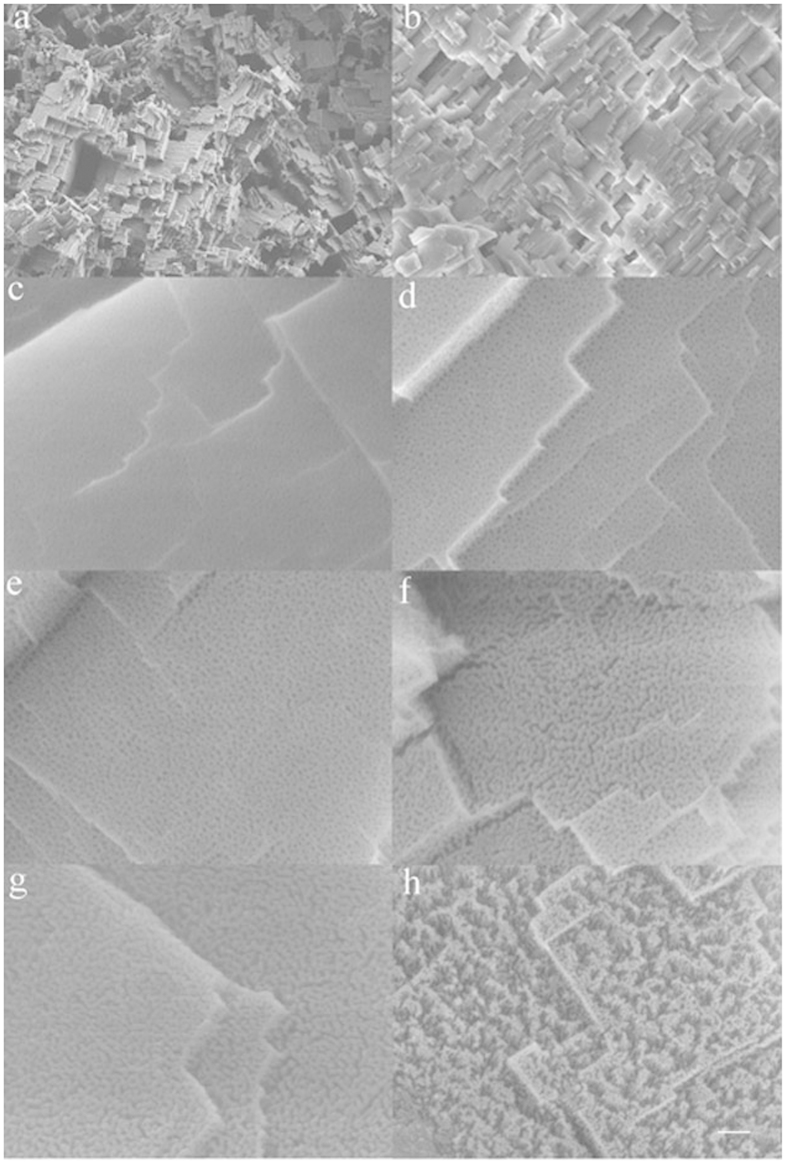
Topographical evolution of the aluminum surface. The low resolution SEM images in **a** and **b** show the morphology after acid etching and after anodization for 20 min, respectively. The high resolution SEM images in (**c–h**) show the detail evolution of the surface at anodization time of 10 min, 15 min, 20 min, 25 min, 30 min, 35 min, respectively. The scale bar represents 8 μm for a, 2 μm for b, 100 nm for **c–g**, and 200 nm for h.

**Figure 3:**
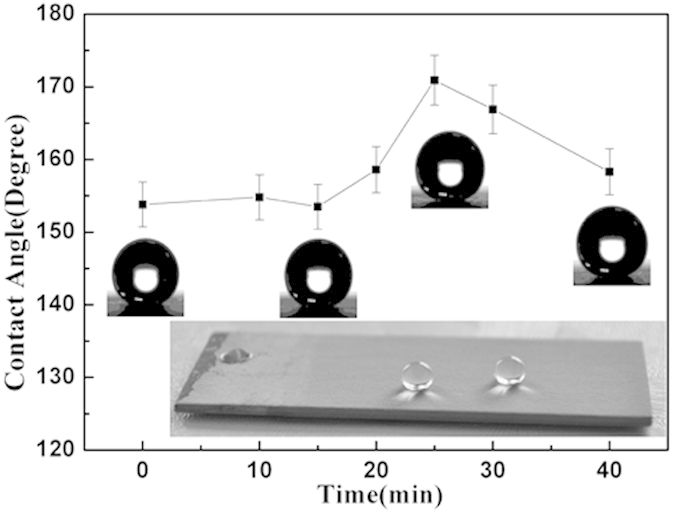
The CA evolution of the SHSs with anodizing time.

**Figure 4:**
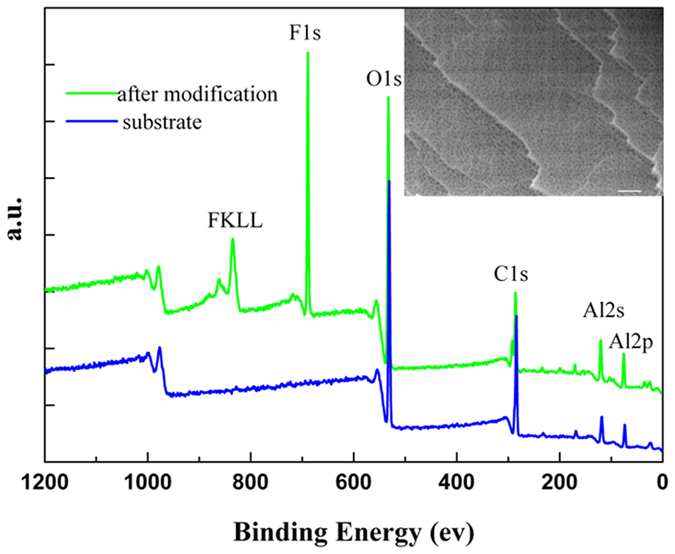
XP survey spectra of the alumina substrate before and after modification. This inset shows the corresponding SEM image after modification.

**Figure 5:**
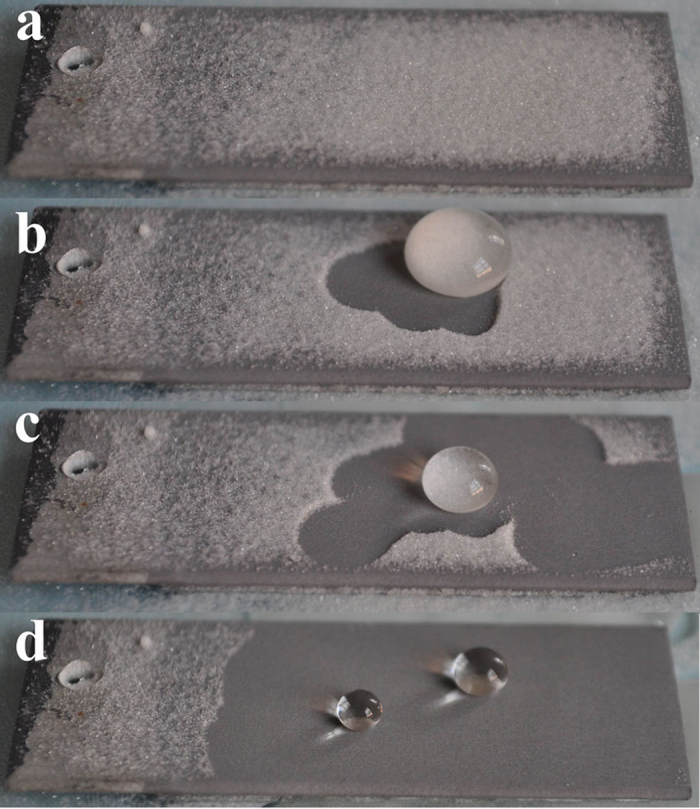
The photographs showing the self-cleaning proceeding during which the covering sands were taken away by sliding water droplets.

The image in Fig. 2a shows the surface morphology after etching in HCl aqueous solution for 11 min. Millions of facets with a dimension below 1 μm build up numerous steps and pits, which is typical etching morphology of aluminum^33^,^36^,^37^. These steps and pits are reserved even after anodizing. A typical SEM image of a sample after anodizing for 20 min is shown in Fig. 2b. The edges of the steps, which are the most sensitive parts in corrosion medium, are as sharp as before anodizing. Detailed study on the surface morphology evolution indicates the nanopores are formed within 10 min (Fig. 2c). The pore density and pore size increase as the anodic oxidation progressed, as show in Fig. 2c-g. In fact, the density and the size reach a maximum value at 25 min. The walls isolating adjacent pores become thinner and thinner as the anodic oxidation progress. At 25 min, some walls began to break up, and the adjacent pores linked together. These linked pores become more and more as proved by the image of Fig. 2g at 30 min. Eventually, all pores are linked together. The residual walls which have already been separated by the linked pores into nanowires congregate into bunch. All the pristine anodized sample surfaces are hydrophilic, because the top layer Al_2_O_3_ can react with H_2_O to generate some hydrophilic functional groups, such as hydroxyl groups. The water droplet is absorbed quickly once it is contacting the surface. But after modification by immersing it in the FDTS-ethanol solution, all the sample surfaces transited to be superhydrophobic. FDTS molecules form self-assembled monolayers. They bond onto the surface terminated with hydroxyl groups forming a regular covalent bond. It anchors on oxide surfaces with its tricholoro-silane group and attaches covalently. Due to its heavily fluorinated tail group which predominantly oriented perpendicular to the substrate surface, an FDTS monolayer reduces surface energy as low as 6.7 mJ/m^2^ 40,41. The CA of each modified sample was carefully measured, and the result was shown in Fig. 3. The insets in Fig. 3 are typical photos of a water droplet on each SHS. All the CA is above 150°, that is to say, all these surfaces are SHS theoretically. Carefully analyzing the results, it is found that the CA firstly increased with anodization time and then decreased. CA got a maximum value on the surface anodized for 25 min, where the highest pore density and largest pore size were obtained. According to Wenzel equation (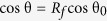, where θ is the CA of a water droplet upon a rough solid surface, θ_0_ is the CA upon a flat solid surface, the non-dimensional surface roughness factor R_*f*_ is the ratio of the surface area, A_SL_, to its flat projected area, A_F_) and Cassie-Baxter equation (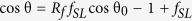, where *f*_SL_ represents the solid-liquid fractional area in the composite interface.), the realistic CA on a rough solid surface,θ, should increase with surface roughness factor R_*f*_. The generations of the nanopores on the facets definitely increase the surface area A_SL_, and then the roughness factor R_*f*_. Thus, the realistic CA will increase with anodic oxidation duration. But the increase of the surface area induced by anodic oxidation has a limitation. Further anodic oxidation will induce linking of pores and congregation of the residual nanowires, which leads to the decrease of the A_SL_. That is why CA has a peak value at 25 min. In the following XPS, mechanical durability, corrosion and ice-phobic experiment, only the sample anodized for 25 min was selected. Figure 4 shows the X-ray photoelectron spectra of the alumina surface before and after modification. The XPS from the alumina surface reveals Al(2p) peak at 76 eV, Al(2 s) peak at 120 eV, C(1 s) peak at 284 eV and O(1 s) peak at 531 eV. After modification, the XPS exhibits two more peaks of F(1 s) at 688.8 eV and F(KLL) at 834.4 eV beside all the peaks appearing in aforementioned XPS results. The XPS results confirm the presence of an FDTS monolayer on the surface after modification. But according to the SEM observation as shown in the inset of Fig. 4, this monolayer have none infection on the morphology. The nanosized pores and steps on the surface are still clear after covering the monolayer. The CA was measured again on the same modified surface after it was kept in air at room temperature for a year. The one-year-aged sample exhibited the same superhydrophobic behavior, indicating that the SHS is reliable in air at room temperature. If titling the sample gently, the water droplet lunges forward and finally slides downward as the tilted angle is close to 1°. That endows the surface self-cleaning ability and the ability is testified by the following experiment. The sample was placed into a culture dish and covered with sand, as show in Fig. 5a. As water droplets poured upon the surface, the sand could be easily swallowed by the droplet, as shown in Fig. 5b,c. When the water droplets bounced off the surface, the swallowed sands were taken away, and the surface became clean, as recorded by the image in Fig. 5d. A similar procedure was recorded by the video of movie-1 in supplementary materials.

**Figure 6:**
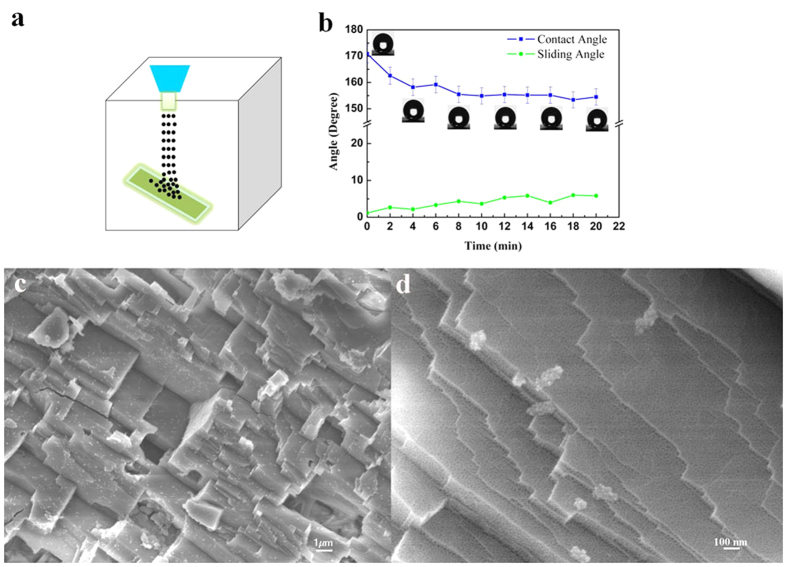
(**a**) Schematic illustration of sand abrasion test employed to evaluate the robustness. (**b**) Evolution of CA and SA with sand abrasion time. (**c,d**) SEM images showing the morphology of the surface after sand abrasion.

**Figure 7:**
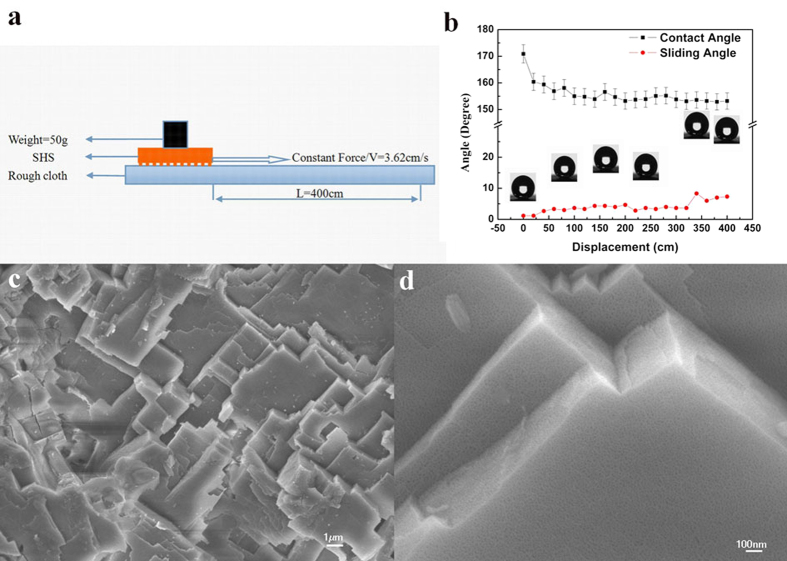
(**a**) Schematic illustration of fabric abrasion test employed to evaluate the robustness. (**b**) Evolution of CA and SA with fabric abrasion time. (**c,d**) SEM images showing the morphology of the surface after fabric abrasion.

Three experiments were designed in order to determine the durability of the SHS. For a Cassie-Baxter droplet, it forms millions of convex shaped menisci between adjacent hydrophobic asperities during static or sliding contact. These menisci develop a positive pressure which supports the droplet standing or sliding. The collapses of these menisci lead to the transition from slippery surface to sticky surface. Firstly, in order to investigate the stability of liquid menisci under long-term exposure to water and water droplets impacting, the surface was continuously sunk in 0.5 m deep underwater for 24 hours and impacted by freely falling water droplets from 20 cm high for 72 hours, as recorded by the movie-2. During that period, 6L water or about 2,40, 000 droplets impacted on the surface. None residual water was observed on the surface at the end of both experiments. It proves that the Cassie-Baxter state of the surface is very stable. According to the Young-Laplace equation 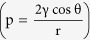, the pressure difference, p, is proportional to the interfacial tension,γ, and inversely proportional to the effective radius, r, of the interface, and it also depends on the wetting angle, θ, of the liquid on the surface of the capillary. The effective radius for the nanopore in present experiment is tiny, around 10 nm. So it can give a very high pressure difference which guarantees the stability of the liquid meniscus on each nanopore and then the stability of the air cushion on the SHS under long-term exposure to water. Secondly, the mechanical durability was testified by sand abrasion, as illustrated by Fig. 6a. The procedure lasted 20 min and was recorded by the movie-3. And the effect on the wettability of the surface was described by Fig. 6b. In the initial 10 min, the CA declined dramatically, from 170° down to about 155°. Then it was almost maintained at this value for the following 10 min. The SA also increased gently from about 1° to 5°, which indicates it still reserved the self-cleaing ability. To prove that, we changed the sample angel to a low value to make sure the falling sand could stand on the sample, and then dropped water on the sample. As recorded by movie-4, the sands were taken away by the sliding water droplets. The morphology after sand abrasion was observed by SEM, and the images are shown in Fig. 6c,d. It is clear that the micrometer scaled asperities almost retained their original morphology except that some edges and some steps which weakly linked to the substrate collapsed partially. For the nanoscale asperities on the sample surface consisted of nanopores which shrunk into the substrate, the sand impacting has very little effect on them, as proved by the SEM image in Fig. 6d. Thirdly, the mechanical durability was testified by the polypropylene fabric abrasion, as illustrated by Fig. 7a. The sample was pressed onto the polypropylene fabric surface by a weight of 50 g and slid on it at a constant speed under manual traction. The wettability evolution during the abrasion process was carefully investigated and the results were shown in Fig. 7b. The results are very consistent with the sand abrasion. The sharp CA drop and SA increase only took place at the initial distance of 150 cm. The CA dropped from 170° to 155° and the SA increased from 1° to 4°. Then the further abrasion did not cause much changing of the CA and SA. After 4 m sliding friction, the CA was about 153°. The SA was below 5° for most of the time before it steeply rose from 4° to 8° in the last 60 cm distance. Thus the excellent self-cleaning ability was well retained after the long distance sliding friction. The SEM images in Fig. 7c,d show the morphology after abrasion. It is clear that the abrasion only caused loss to small tall steps which protruded from the surface. The morphology of the pits was well protected as well as the nanopores, as shown in Fig. 7d.

**Figure 8:**
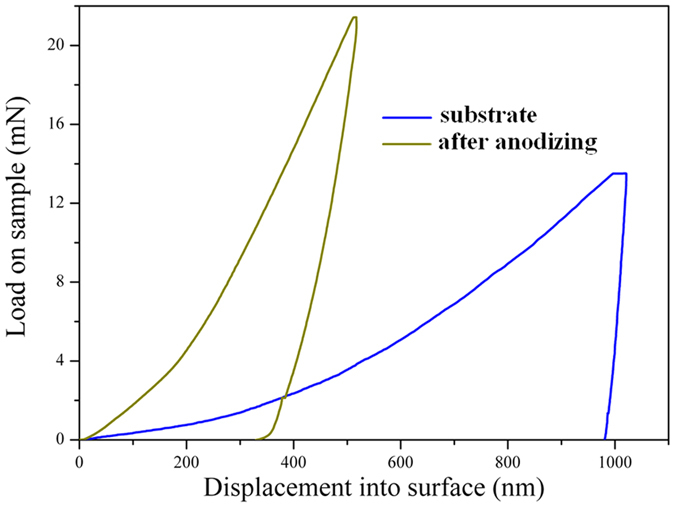
Nanoindentation load-displacement curves of commercially pure Al substrate and that after anodization, which indicates the hardness of the top surface increased sharply after anodization.

The excellent mechanical durability of the surface should be relevant to the very high hardness of the Al_2_O_3_ coating which was induced by the anodization process. The hardness of the samples before and after anodization was both carefully investigated by nanoindentation. And the results are shown in Fig. 8. The hardness of Al substrate is only 0.5 Gpa. But the hardness after anodization steeply rises six times to 4 Gpa as against Al substrate because of the Al_2_O_3_ coating. The sand particle is very huge compared to the steps. As it drops on the surface, it should cover numerous steps. In fact, a step which is punched by a falling sand particle just suffers a very little impact. That is not enough to cause large deformation or damage on the step because of the ultrahigh hardness. For the SHSs which mimic Lotus leaves, the nanoscale asperities are always the most fragile parts to mechanical abrasion. But in our experiment, they become the safest parts because they are protected by the ultra-hard Al_2_O_3_ coating. Thus the morphology can remain almost unchanged after millions of impacts by sand particles. The slightly fading of the self-cleaning abilities may be related to the damage of steps which generates fresh hydrophilic surfaces. For the fabric abrasion experiment, the high hardness of the coating makes sure the steps protruding from the surface has enough strength to support the weight and prevent the pits and nanopores from contacting the fabric. Thus after abrasion, only the tall protruding steps are truncated. Of cause, the FDTS monolayers on the top surface of the steps are also lost. That induces the initial sharp drop of CA and increase of SA for fabric abrasion. But for the following abrasion, the friction just happen between the fabric and the truncated steps, which would not further damage the superhydrophobicity. Thus, the CA and SA are maintained at a constant value for following friction.

**Figure 9:**
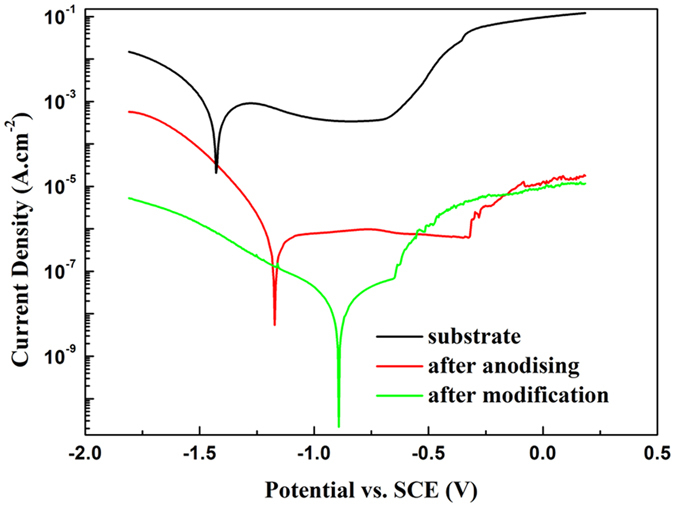
Potentiodynamic curves of commercially pure Al substrate and anodized substrate and SHS.

We investigated the corrosion resistance of the SHS in 3.5 wt% NaCl aqueous solution from the electrochemical points of view. Figure 9 shows potentiodynamic polarization curves of Al substrate and the anodized sample and the SHS. As compared to the corrosion current density of the Al substrate (1.65 ×10^−4 ^A/cm^2^), that of the anodized sample (2.42 ×10^−6 ^A/cm^2^) decreases by more than 2 order of magnitude. The surperhydrophobic surface shows the lowest corrosion current density (2.36 ×10^−8 ^A/cm^2^) which decreases by more than 4 order of magnitude than the Al substrate. As the Al substrate in our experiment is etched by HCl solution, the area of the true interface with NaCl aqueous solution, in fact, is much more than 1 cm^2^ meanwhile the natural oxide film forming on Al surface has been partially destroyed. Thus the corrosion current density and the corrosion potential (−1.43 V) of the Al substrate is higher and more negative than other reports^42^. The anodization process is really helpful to improve the corrosion resistance, which endow the sample a very low corrosion current density and shift the corrosion potential to more positive position (−1.17 V). It should be noted that the SHS owes the lowest corrosion current density and the most positive corrosion potential (−0.88 V), which indicates that electrochemical corrosion is very difficult to happen on such surface. Even if it takes place, it proceeds at a very low rate. It supports the conclusion that the superhydrophobic treatment is effective for improving the corrosion resistance. For the SHS, there is an air cushion separating the substrate from the NaCl aqueous solution, which induces a very less realistic contacting area between the substrate and electrolyte, and thus should be beneficial to enhancing the corrosion resistance and lowering the corrosion current. Hydrogen evolution dominates at more negative potentials than corrosion potential, resulting in an increase in the cathodic currents. At more positive potentials than corrosion potential, oxidation prevails which induces surface passivation and a flat stage in each curve. Apparently, the SHS has a much shorter stage than the anodized sample, which should be attributed to the disturbance of the air cushion.

**Figure 10:**
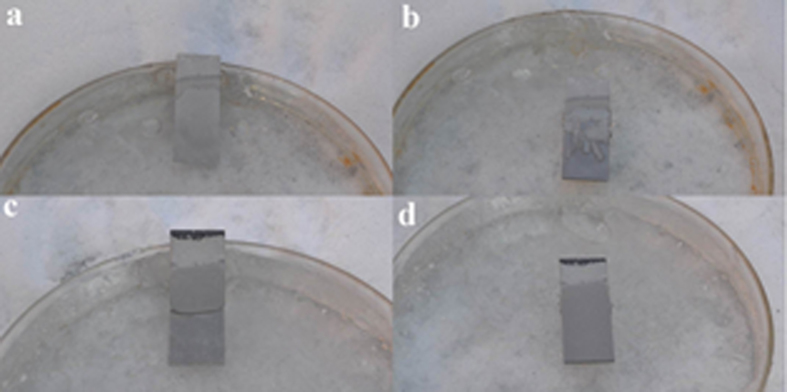
The comparison of surface de-icing property between anodized substrate(up) and SHS(down). The frozen states of the anodized substrate and SHS were shown in (**a,c**) respectively. And the states after drawing out were shown in b and d repectively.

The anti-icing properties of the SHS were investigated by a similar experiment to the movie-2, but it was completed at ambient temperature of −20°C. As the supercooled water poured down onto the subzero surface, the droplets timely rebounded off before freezing on the SHS. We carefully set a water droplet on the SHS. It was frozen after a while. The transition really increased the adhesion which inhibited it shed off by gravity. But the adhesion force was much lower than the anodized sample. In order to emphasize this effect, an anodized sample and a SHS both immerged into water at ambient temperature of −20°C. Half an hour later, the water was frozen and it crusted the samples, as shown by Fig. 10. It was very hard to pull out the anodized sample only when the ice was fractured. After pulled out, as shown by Fig. 10b, there was still a piece of ice attached on the surface. But it was very easy to pull out the superhydrophobic sample. And after pulled out, the surface was very clean, none of ice attaching on it. The pristine anodized sample is hydrophilic. After immerging into water, the water, and then the ice, that has saturated the surface asperities can create a tight bond with the surface, increasing the strength of ice adhesion. But for the surperhydrophobic surface, the air cushion seals the nanopores and separates most of the substrate surface from attachment to the ice. Thus it can be easily pulled out from the ice and get rid of ice adhesion. The porous structure endows the sample a potential anti-icing property by gravity. As shown in movie-5, the SHS was tilted to a degree of 10 relative to horizontal line and a block of ice was set on the top of the surface. The ice was immobile initially because of the friction force. After the ice was melt partially, a water layer formed between the surface and the ice which facilitated the sliding by gravity.

## Concluding Remarks

SHSs were successfully fabricated on etched commercially pure aluminum substrate through the method of anodization combining modification by FDTS. Ascribed to the ultrahigh hardness of Al_2_O_3_ coating induced by anodization and the nanopores instead of the fragile nanoscale protrusions which are essential for SHSs mimicking Lutos leaves, the surfaces exhibited excellent durability as demonstrated by long term exposure to water impacting, cycles of sand abrasion and fabric abrasion. The surfaces also had good corrosion resistance and ice-phobicity. For fabrication method is scalable, these self-cleaning surfaces are anticipated to have important practical applications on body panels of automobiles and aircrafts.

## Additional Information

**How to cite this article**: Wang, G. *et al.* Robust superhydrophobic surface on Al substrate with durability, corrosion resistance and ice-phobicity. *Sci. Rep.* **6**, 20933; doi: 10.1038/srep20933 (2016).

## Supplementary Material

Supplementary Information

Supplementary Movie 1

Supplementary Movie 2

Supplementary Movie 3

Supplementary Movie 4

Supplementary Movie 5

## Figures and Tables

**Figure 1:**
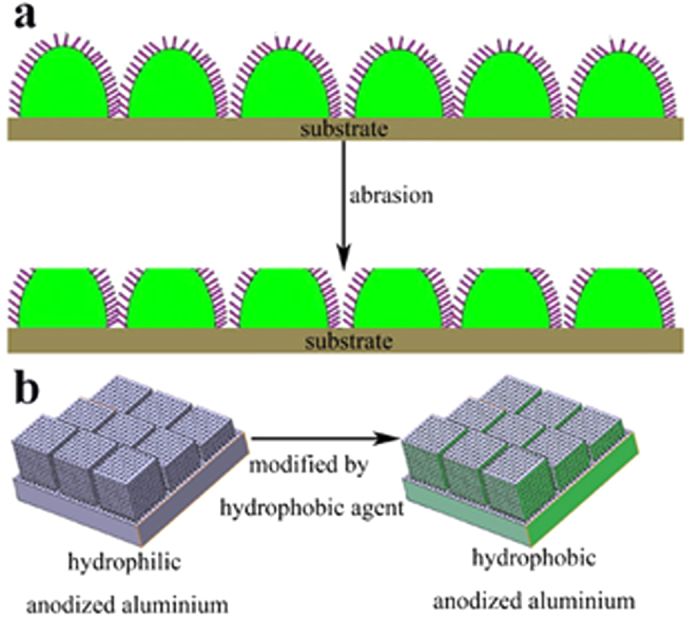
Schematic illustration of the methodology to solve the problem caused by mechanical damage. (**a**) The microscopic asperities with micrometers suffer the wear and most of the surface morphology is protected by them. (**b**) Schematic depiction of the approach used in this paper where nanopores appear instead of nanoscale protrusions.

## References

[b1] Bhushan B. & Jung Y. C. Natural and biomimetic artificial surfaces for superhydrophobicity, self-cleaning, low adhesion and drag reduction. Prog. Mater Sci. 56, 1–108 (2011).

[b2] Yao L. & He J. Recent progress in antireflection and self-cleaning technology - From surface engineering to functional surfaces. Prog. Mater. Sci. 61, 94–143 (2014).

[b3] Sahoo B. N. & Kandasubramanian B. Recent progress in fabrication and characterisation of hierarchical biomimetic superhydrophobic structures. Rsc Adv. 4, 22053–22093 (2014).

[b4] Rioboo R., Delattre B., Duvivier D., Vaillant A. & De Coninck J. Superhydrophobicity and liquid repellency of solutions on polypropylene. Adv. Colloid Interface Sci. 175, 1–10 (2012). 22483352 10.1016/j.cis.2012.03.003

[b5] Celia E., Darmanin T., de Givenchy E. T., Amigoni S. & Guittard F. Recent advances in designing superhydrophobic surfaces. J. Colloid Interface Sci. 402, 1–18, (2013). 23647693 10.1016/j.jcis.2013.03.041

[b6] Guo P., Wen M., Wang L. & Zheng Y. Strong anti-ice ability of nanohairs over micro-ratchet structures. Nanoscale 6, 3917–3920 (2014). 24122128 10.1039/c3nr04061e

[b7] Davis A., Yeong Y. H., Steele A., Bayer I. S. & Loth E. Superhydrophobic Nanocomposite Surface Topography and Ice Adhesion. ACS Appl. mater. Interfaces 6, 9272–9279 (2014). 24914617 10.1021/am501640h

[b8] Toma M., Loget G. & Corn R. M. Flexible Teflon Nanocone Array Surfaces with Tunable Superhydrophobicity for Self-Cleaning and Aqueous Droplet Patterning. ACS Appl. Mater. Interfaces 6, 11110–11117 (2014). 24654844 10.1021/am500735v

[b9] Wu Y., Hang T., Yu Z., Xu L. & Li M. Lotus leaf-like dual-scale silver film applied as a superhydrophobic and self-cleaning substrate. Chem. Commun. 50, 8405–8407, (2014). 10.1039/c4cc03878a 24946911

[b10] Hong D. *et al.* Mussel-inspired, perfluorinated polydopamine for self-cleaning coating on various substrates. Chem. Commun. 50, 11649–11652 (2014). 10.1039/c4cc02775b 24946130

[b11] Koch K., Bhushan B., Jung Y. C. & Barthlott W. Fabrication of artificial Lotus leaves and significance of hierarchical structure for superhydrophobicity and low adhesion. Soft Matter 5, 1386–1393 (2009).

[b12] Liu K. & Jiang L. Metallic surfaces with special wettability. Nanoscale 3, 825–838 (2011). 21212900 10.1039/c0nr00642d

[b13] Ozcan O., Wang H., Taylor J. D. & Sitti M. STRIDE II: A Water Strider-inspired Miniature Robot with Circular Footpads. Int. J. Adv. Rob. Syst. 11, 85 (2014).

[b14] Dash S., Chandramohan A., Weibel J. A. & Garimella S. V. Buoyancy-induced on-the-spot mixing in droplets evaporating on nonwetting surfaces. Phys. Rev. E 90, 062407 (2014). 10.1103/PhysRevE.90.062407 25615112

[b15] Choi Y. *et al.* Beauty of Lotus is More than Skin Deep: Highly Buoyant Superhydrophobic Films. ACS Appl. Mater. Interfaces 6, 7009–7013 (2014). 24801001 10.1021/am5015343

[b16] Wang P., Zhang D., Qiu R. & Wu J. Super-hydrophobic metal-complex film fabricated electrochemically on copper as a barrier to corrosive medium. Corros. Sci. 83, 317–326, (2014).

[b17] Zang D., Zhu R., Wu C., Yu X. & Zhang Y. Fabrication of stable superhydrophobic surface with improved anticorrosion property on magnesium alloy. Scr. Mater. 69, 614–617, (2013).

[b18] Zhu X. *et al.* A versatile approach to produce superhydrophobic materials used for oil-water separation. J. Colloid Interface Sci 432, 105–108, (2014). 25086383 10.1016/j.jcis.2014.06.056

[b19] Tang X. *et al.* *In situ* polymerized superhydrophobic and superoleophilic nanofibrous membranes for gravity driven oil-water separation. Nanoscale 5, 11657–11664 (2013). 24100352 10.1039/c3nr03937d

[b20] Li X., Hu D., Huang K. & Yang C. Hierarchical rough surfaces formed by LBL self-assembly for oil-water separation. J. Mater. Chem. A 2, 11830–11838 (2014).

[b21] Lu Y. *et al.* Creating superhydrophobic mild steel surfaces for water proofing and oil-water separation. J. Mater. Chem. A 2, 11628–11634, (2014).

[b22] Shillingford C., MacCallum N., Wong T.-S., Kim P. & Aizenberg J. Fabrics coated with lubricated nanostructures display robust omniphobicity. Nanotechnology 25, 014019 (2014). 24334333 10.1088/0957-4484/25/1/014019

[b23] Zhu Q., Gao Q., Guo Y., Yang C. Q. & Shen L. Modified Silica Sol Coatings for Highly Hydrophobic Cotton and Polyester Fabrics Using a One-Step Procedure. Ind. Eng. Chem. Res. 50, 5881–5888 (2011).

[b24] Xue C.-H. & Ma J.-Z. Long-lived superhydrophobic surfaces. J. Mater. Chem. A 1, 4146–4161 (2013).

[b25] Burton Z. & Bhushan B. Hydrophobicity, adhesion and friction properties of nanopatterned polymers and scale dependence for micro- and nanoelectromechanical systems. Nano Lett. 5, 1607–1613, (2005). 16089497 10.1021/nl050861b

[b26] Extrand C. W. & Moon S. I. Repellency of the Lotus Leaf: Contact Angles, Drop Retention and Sliding Angles. Langmuir 30, 8791–8797 (2014). 25029189 10.1021/la5019482

[b27] Wang G. Y. & Zhang T. Y. Easy Route to the Wettability Cycling of Copper Surface between Superhydrophobicity and Superhydrophilicity. ACS Appl. Mater. Interfaces 4, 273–279 (2012). 22148586 10.1021/am2013129

[b28] Wang G. Y. & Zhang T. Y. Oxygen adsorption induced superhydrophilic-to-superhydrophobic transition on hierarchical nanostructured CuO surface. J. Colloid Interface Sci. 377, 438–441, (2012). 22484170 10.1016/j.jcis.2012.03.035

[b29] Kondrashov V. & Ruhe J. Microcones and Nanograss: Toward Mechanically Robust Superhydrophobic Surfaces. Langmuir 30, 4342–4350 (2014). 24628022 10.1021/la500395e

[b30] Ishizaki T., Masuda Y. & Sakamoto M. Corrosion Resistance and Durability of Superhydrophobic Surface Formed on Magnesium Alloy Coated with Nanostructured Cerium Oxide Film and Fluoroalkylsilane Molecules in Corrosive NaCl Aqueous Solution. Langmuir 27, 4780–4788 (2011). 21417352 10.1021/la2002783

[b31] Wang N., Xiong D. S., Deng Y. L., Shi Y. & Wang K. Mechanically Robust Superhydrophobic Steel Surface with Anti-Icing, UV-Durability and Corrosion Resistance Properties. ACS Appl. Mater. Interfaces 7, 6260–6272 (2015). 25749123 10.1021/acsami.5b00558

[b32] Jung Y. C. & Bhushan B. Mechanically Durable Carbon Nanotube-Composite Hierarchical Structures with Superhydrophobicity, Self-Cleaning, and Low-Drag. Acs Nano 3, 4155–4163 (2009). 19947581 10.1021/nn901509r

[b33] Maitra T. *et al.* Hierarchically nanotextured surfaces maintaining superhydrophobicity under severely adverse conditions. Nanoscale 6, 8710–8719 (2014). 24947006 10.1039/c4nr01368a

[b34] Zhu X. T. *et al.* Robust superhydrophobic surfaces with mechanical durability and easy repairability. J. Mater. Chem. 21, 15793–15797 (2011).

[b35] Xiu Y., Liu Y., Hess D. W. & Wong C. P. Mechanically robust superhydrophobicity on hierarchically structured Si surfaces. Nanotechnology 21, 155705 (2010). 20332558 10.1088/0957-4484/21/15/155705

[b36] Wu W. *et al.* Alumina nanowire forests via unconventional anodization and super-repellency plus low adhesion to diverse liquids. Chem. Comm. 45, 1043–1045 (2009). 19225630 10.1039/b818633b

[b37] Wang X., Liu X., Zhou F. & Liu W. Self-healing superamphiphobicity. Chem. Comm. 47, 2324–2326 (2011). 21152663 10.1039/c0cc04066e

[b38] Wang D. *et al.* Towards a tunable and switchable water adhesion on a TiO2 nanotube film with patterned wettability. Chem. Comm. 45, 7018–7020 (2009). 19904380 10.1039/b914630j

[b39] Yildirim A. *et al.* Superhydrophobic and Omnidirectional Antireflective Surfaces from Nanostructured Ormosil Colloids. ACS Appl mater. Interfaces 5, 853–860 (2013). 23281919 10.1021/am3024417

[b40] Schondelmaier D. *et al.* Orientation and Self-Assembly of Hydrophobic Fluoroalkylsilanes. Langmuir 18, 6242–6245 (2002).

[b41] Sharpe R. B. A. *et al.* Chemically Patterned Flat Stamps for Microcontact Printing. J. Am. Chem. Soc. 127, 10344–10349 (2005). 16028946 10.1021/ja052139l

[b42] Kazemi M., Danaee I. & Zaarei D. The effect of pre-anodizing on corrosion behavior of silicate conversion coating on AA2024. Mater. Chem. Phys. 148, 223–229 (2014).

