# Supplementary material for: Robust superhydrophobic surface on Al substrate with durability, corrosion resistance and ice-phobicity
Source: Sci Rep. 2016 Feb 8;6:20933. doi: 10.1038/srep20933 (PMC4745080; doi:10.1038/srep20933)
Supplement: Supplementary Information [file srep20933-s1.pdf]

Robust superhydrophobic surface on Al substrate with durability, corrosion resistance and ice-phobicity

Guoyong Wang[a*], Shuai Liu[a], Sufeng Wei[b], Yan Liu [c*], Jianshe Lian[a], Qing Jiang[a]

[a] Key Laboratory of Automobile Materials, Department of Materials Science and Engineering, Jilin University, No. 5988 Renmin Street, Changchun 130025, PR China

[b]Key Laboratory of Advanced Structural Materials, Changchun University of Technology, Changchun 130012, PR China

[c]Key Laboratory of Bionic Engineering (Ministry of Education), Jilin University, Changchun 130022, China

Video legends:

Movie-1:The self-cleaning ability of the SHS. The SHS is covered by sand. As water is pouring down onto the surface, the sand is swallowed and taken away by the bouncing water droplets. That validates the self-cleaning ability of the SHS.

Movie-2: Water droplets impacting experiment to investigate the stability of the self-cleaning surface. During this experiment, 6L water freely falls from 20 cm high onto the surface in order to check whether the surface would be wetted under durative water droplets impacting.

Movie-3: Sand abrasion experiment on the SHS. Sands with diameter ranging from 120 μm to 260 μm flow down onto the surface at a speed of 9 g/min from a height of 30cm for 20 min. The SHS keeps an appropriate angle to make sure the sands can slide off the surface freely.

Movie-4: The self-cleaning experiment after sand abrasion.

Movie-5: Deicing experiment. A block of ice was covered on the surface. In order to get rid of it, we heated the surface. After the ice was melt partially, a water layer formed between the surface and the ice which facilitated the sliding by gravity. Thus, it is not necessary to melt all the block of ice.
